# Horizontal Plasmid Transfer by Transformation in *Escherichia coli*: Environmental Factors and Possible Mechanisms

**DOI:** 10.3389/fmicb.2018.02365

**Published:** 2018-10-04

**Authors:** Haruka Hasegawa, Erika Suzuki, Sumio Maeda

**Affiliations:** Graduate School of Humanities and Sciences, Nara Women’s University, Nara, Japan

**Keywords:** plasmid transformation, horizontal plasmid transfer, *Escherichia coli*, antibiotic resistance, solid-air biofilm

## Abstract

Transformation is one mode of horizontal gene transfer (HGT) in bacteria, wherein extracellular naked DNA is taken up by cells that have developed genetic competence. Sensitivity to DNase, which degrades naked DNA, is the key to distinguishing transformation from the DNase-resistant HGT mechanisms. In general, *Escherichia coli* is not believed to be naturally transformable; it develops high competence only under artificial conditions, including exposure to high Ca^2+^ concentrations. However, *E. coli* can reportedly express modest competence under certain conditions that are feasible in natural environments outside laboratory. In addition, recent data suggest that environmental factors influence multiple routes of transformation. In this mini review, we (1) summarize our studies on transformation-based HGT using *E. coli* experimental systems and (2) discuss the possible occurrence of transformation via multiple mechanisms in the environment and its possible impact on the spread of antibiotic resistance genes.

## Introduction

Horizontal gene transfer (HGT) between bacterial cells contributes to bacterial adaptation to various environments and, in the long term, to bacterial evolution (Lorenz and Wackernagel, 1994; Bushman, 2002; Thomas and Nielsen, 2005). However, in human environments, it causes undesirable spread of pathogenicity, antibiotic resistance, or artificially engineered genes (Bushman, 2002; Keese, 2008; Kelly et al., 2009a,b). Three mechanisms of HGT in bacteria are generally accepted: conjugation, transduction, and transformation (Bushman, 2002; von Wintersdorff et al., 2016). Conjugation and transduction involve specific apparatus for DNA transfer from donor to recipient cells; these are conjugative pili and phage virions, respectively. Transformation is primarily a function of recipient cells that express competence to take up extracellular naked DNA.

Transformation competence can be naturally or artificially induced, but not all bacterial species develop natural competence (Lorenz and Wackernagel, 1994; Johnston et al., 2014). In naturally transformable bacteria, competence is usually transient and induced by alterations in the growth state of organism (Johnston et al., 2014). A group of “competence genes” has been identified, and general mechanistic models have been proposed (Chen and Dubnau, 2004), although precise mechanisms for individual bacterial species have not been sufficiently elucidated (Cameron and Redfield, 2006, 2008; Sinha et al., 2009; Seitz and Blokesch, 2013; Johnston et al., 2014; Jaskólska and Gerdes, 2015). Because transformation requires extracellular naked DNA as the substrate, sensitivity to DNase, which degrades naked DNA, is key in distinguishing transformation from other DNase-resistant HGT mechanisms (Lorenz and Wackernagel, 1994; Giovanetti et al., 2005; Marshall et al., 2010; Rohrer et al., 2012; Blesa and Berenguer, 2015).

In general, *Escherichia coli* is not believed to be naturally transformable; it develops high genetic competence only under artificial conditions, including exposure to high Ca^2+^ concentrations and temperature shock (Mandel and Higa, 1970; Hanahan, 1983; Sambrook et al., 1989), polyethylene glycol treatment (Chung et al., 1989; Sambrook et al., 1989), or electrical shock (Sambrook and Russell, 2006). However, reportedly, *E. coli* can express modest competence under certain conditions that are feasible in its natural environments (Baur et al., 1996, Bauer et al., 1999; Tsen et al., 2002; Woegerbauer et al., 2002). In the following, we define transformation wherein plasmid was added externally as plasmid transformation (PT) and transformation wherein plasmid DNA comes from dead bacterial cells (from the environment) as horizontal plasmid transfer by transformation (HPTT).

*Escherichia coli* seems to possess multiple DNA-uptake mechanisms, including two popular ones: one that is dependent on the “competence genes,” which commonly work in many gram-negative and -positive bacteria (Finkel and Kolter, 2001; Palchevskiy and Finkel, 2006; Sinha et al., 2009; Sinha and Redfield, 2012; Seitz and Blokesch, 2013; Johnston et al., 2014; Jaskólska and Gerdes, 2015). This mechanism is mainly conducted by the specific molecular apparatus formed around the cell surface structure, which pass through the cell membranes only linear single-stranded DNA produced using a specific periplasmic nuclease. In *E. coli*, these genes are not considered to contribute to PT because PT requires the uptake of intact double-stranded circular DNA (Sinha and Redfield, 2012; Johnston et al., 2014). Therefore, it is unlikely that this mechanism contributes to PT in the environment. The second mechanism is that dependent on external environmental factors, such as divalent metal ions, heat shock, and physical stresses (Mandel and Higa, 1970; Hanahan, 1983; Yoshida, 2007; Rodríguez-Beltrán et al., 2013). These stimuli are commonly considered to induce the formation of pore-like structures in cell surface for the passing of intact double-stranded DNA, including circular plasmids, although the details remain unclear (Reusch et al., 1986; Reusch and Sadoff, 1988; Huang and Reusch, 1995; Sun et al., 2013; Asif et al., 2017). Ca^2+^ and Mg^2+^ ions are the most typical competence-inducing factors. Environmental habitats often contain several millimolar of these ions, whose concentrations are sufficient to induce weak but detectable competence in *E. coli* (Baur et al., 1996, Bauer et al., 1999; Maeda et al., 2003). Therefore, this mechanism is possible in the environment outside laboratories. In addition to the above two mechanisms, another mechanism has been proposed by Sun et al. (2006, 2009), Zhang et al. (2012), Guo et al. (2015), and Sun (2016), in which an ABC transporter and specific periplasmic and inner membrane proteins are involved. This mechanism is regulated by internal transcriptional regulators, RpoS and CRP, therefore it was suggested that this mechanism is also a genetically controlled natural process.

In this mini review, we summarize our studies on HGT using *E. coli* experimental systems and discuss the possible occurrence of transformation by multiple mechanisms in natural environments and its possible impact on the spread of antibiotic resistance genes.

## Plasmid Transformation of *E*. *coli* in Conditions Mimicking Natural Environment

### PT in Food Extracts

Human foods are excellent culture media for many bacteria. However, little attention has been paid to the effects of foods on bacterial physiology other than growth and survival. We investigated the possibility that foods act as media for bacterial transformation. Foods often contain millimolar concentrations of divalent metal ions (Ca^2+^ and Mg^2+^) and are often stored in a refrigerator or freezer followed by rapid warming (i.e., heat shocked). These conditions are conducive to the development of competency in *E. coli* (Mandel and Higa, 1970; Huang and Reusch, 1995; Baur et al., 1996); because *E. coli* is a common food contaminant, it is interesting to determine whether it can be transformed in foods. Certain foods can indeed act as media that induce competency in *E. coli* (Maeda et al., 2003). Of 42 food samples tested, >10 exhibited an ability to induce competency at a frequency of 10^−7^−10^−9^. Among these, the supernatant from tofu (a cheese-like food made of curdled soybean milk) exhibited the highest activity (one in 10^−7^−10^−8^ recipient cells), corresponding to approximately one-half of the efficiency obtained with 100 mM CaCl_2_. However, there were no clear correlations between transformation frequencies and chemical characteristics of the foods (Ca^2+^ or Mg^2+^ concentrations and pH), suggesting that complex factors within the foods affect competency development. Similar effects of foods in inducing transformation have been reported in *E. coli* (Bauer et al., 1999) and *Bacillus subtilis* (Brautigam et al., 1997; Zenz et al., 1998).

### PT in Solid-Air Biofilm

Many bacteria exist as biofilms in natural and artificial environments (Davey and O’Toole, 2000). Biofilms are aggregates of microbes that form at solid-liquid or solid-air (SA) interfaces (Anderl et al., 2000; Carmen et al., 2004). Cells in these high-density cultures interact with one another and express distinctive physiological functions compared with their free planktonic forms. Previous studies on *E. coli* transformation exclusively focused on planktonic cells (Mandel and Higa, 1970; Hanahan, 1983), but we showed that *E. coli* cells within SA biofilms develop competence at a frequency of 10^−6^−10^−8^ on various solid media, including LB and H_2_O agar and various moist foods (Maeda et al., 2004). Living cells generally coexist with dead cells in biofilms, and the latter can release their DNA and certain divalent metal ions, including Ca^2+^ and Mn^2+^, into the local microenvironment of the biofilm (Davey and O’Toole, 2000; Whitchurch et al., 2002). These conditions may be conducive for the development of transformation and may not be exclusive to SA biofilms since a similar enhancement in *E. coli* air-liquid biofilms has also been reported (Król et al., 2011).

### PT of Wild *E. coli* Strains in Water

Our and others’ results suggest that environmental *E. coli* can potentially acquire foreign DNA via transformation. However, there are few previous reports of investigations into the transformability of natural *E. coli* strains (Woegerbauer et al., 2002; Sinha and Redfield, 2012). Therefore, we examined the potential of natural *E. coli* strains to develop competence under environmental conditions. We used a standard *E. coli* collection of reference (ECOR) strains as our model of natural *E. coli* (Ochman and Selander, 1984) because these ECOR strains have been widely used in various studies on the physiology, behavior, and genotypic variation of natural *E. coli* (Tenaillon et al., 2010). We found that some ECOR strains exhibited detectable transformability (10^−10^−10^−11^) in natural water (commercially available bottled natural pure water) at constant and varying temperatures between 5 and 35°C and at winter temperatures in a field experiment, suggesting that natural *E. coli* can potentially develop competence under certain conditions that could feasibly occur in the environment (Matsumoto et al., 2016b).

## Horizontal Plasmid Transfer by Transformation in *E. coli*

### Freeze–Thaw-Induced HPTT in Natural Waters and Food Extracts

In the environment, naked DNA can be naturally supplied from dead cells to neighboring cells within the same habitat or microenvironment. Therefore, it is worth investigating the possibility of HPTT in a closed system under some feasible conditions. Freeze–thaw is a common process in the handling of foodstuffs and also occurs in nature. Freeze–thaw treatment of *E. coli* cells may promote DNA leakage from dead cells and subsequent uptake by surviving cells because they respond to heat shock, resulting in *in situ* transformation (Li et al., 1992; Takahashi et al., 1992). This treatment of condensed suspensions of mixed *E. coli* strains in natural waters and food extracts caused *in situ* lateral transfer of non-conjugative plasmids at a frequency of 10^−8^−10^−10^ (Ishimoto et al., 2008). This phenomenon also occurred even after 1–2 months of storage at −20°C, and its sensitivity to DNase demonstrated that it was mediated via a transformation mechanism.

### Low Frequency of HPTT in SA Biofilms

Biofilms are thought to be suitable environments for *in situ* transformation because living and dead cells coexist in close proximity, and DNA released from dead cells often accumulates around living cells. In addition, as described above, because *E. coli* cells can develop modest competence in SA biofilms (Maeda et al., 2004), both these factors contribute to HPTT in biofilms. By simply co-culturing a plasmid-free strain with one harboring a non-conjugative plasmid in a SA biofilm on antibiotic-free agar media, transformed cells were produced at low frequency (10^−9^−10^−10^) within 24–48 h (Maeda et al., 2006). Liquid cultures of the same strains in LB broth produced no or few transformants, suggesting the importance of SA biofilm formation for plasmid transfer. Essentially, the same phenomenon occurred in SA biofilms on food-based media (Ando et al., 2009). This phenomenon also occurred between popular laboratory strains such as DH5, HB101, and MG1655 (Etchuuya et al., 2011), which are lysogenic phage-free and conjugative apparatus-free, suggesting that the low frequency of horizontal plasmid transfer in SA biofilms can occur without the aid of phage or conjugation machinery and, therefore, that this DNA transfer is due to a kind of transformation. However, since *rpoS*^−^ mutation did not affect this HPTT (Maeda et al., 2006), the RpoS-dependent mechanism (Zhang et al., 2012) is unlikely to be involved.

### High Frequency of HPTT Induced by P1 Phage

By assessing combinations of several strains and plasmids for horizontal plasmid transfer, the *E. coli* strain CAG18439 was found to act as both a plasmid donor and a plasmid recipient in combination with the plasmid pHSG299 and could frequently transfer the plasmid in a mixed cell culture even in a liquid medium (Etchuuya et al., 2011). This HGT was demonstrated to be a type of transformation because the high frequency plasmid transfer (10^−5^−10^−8^) was DNase-sensitive. Further studies revealed that this phenomenon exhibits some specific characteristics: (1) promotion by proteinaceous factor released from CAG18439 (Etchuuya et al., 2011); (2) promotion by an 88-bp sequence on pHSG299 (Sobue et al., 2011); (3) high transfer frequency (Etchuuya et al., 2011; Sobue et al., 2011); and (4) dependence on specific genes (Kurono et al., 2012; Matsuda et al., 2012). With respect to (1), a later study revealed that these proteinaceous factors include a P1*vir* phage particle (or a derivative thereof) and that externally added P1*vir* phage can reproduce horizontal plasmid transfer between *E. coli* cells and the three other major features of CAG18439-dependent HPTT (Sugiura et al., 2017). This phenomenon was also largely DNase-sensitive, suggesting that a large part of this plasmid transfer is due to transformation despite the involvement of P1 phage. The transformation mechanism of P1*vir* phage-induced plasmid transfer may be due to phage infection or spontaneous awakening of lysogenized phage in plasmid-harboring cells, leading to cell lysis and subsequent intracellular plasmid DNA release in a usable form for transformation. Although such a mechanism is generally feasible, there have been few clear demonstrations of it in *E. coli*. A recent study by Keen et al. (2017) using other phage system also demonstrated a similar phage-induced transformation mechanism in *E. coli*. However, HPTT by P1*vir* or CAG18439 cannot be adequately explained only by enhanced DNA supply from phage-induced cell lysis, and it differs from simple transformation in *E. coli* (Hanahan, 1983) in terms of its distinctive characteristics (2–4). With respect to (2), the 88-bp sequence on pHSG299 is not homologous to the part of the P1 phage genome sequence. This sequence is often found in databases among general cloning vector sequences but not in any natural source. By tracing back the construction process of pHSG299 (Hashimoto-Gotoh et al., 1981; Brady et al., 1984; Takeshita et al., 1987), however, we suspect that the 88-bp sequence originates from R6-5, a conjugative R plasmid. This sequence, and similar DNA elements, may contribute to HPTT of R and other plasmids in the environment. With respect to (3), this high-frequency transfer cannot be explained by the simple PT ability of CAG18439 and other strains used because simple PT in those strains under the equivalent culture condition was 10^5^–10^2^ times less frequent (Etchuuya et al., 2011). It was, therefore, suggested that a CAG18439-derived proteinaceous factor, with size estimated between 9 and 30 kDa (Etchuuya et al., 2011) could also be involved in promoting HPTT. This factor presumably assists in DNA uptake by recipient cells, probably in combination with the 88-bp sequence on the transforming DNA. Lastly, with respect to (4), later genome-wide screening studies for recipient genes involved in HPTT suggested that multiple genes participate in the mechanism (Kurono et al., 2012; Matsuda et al., 2012; Shibata et al., 2014a). These include those that have not been reported to be involved in natural or artificial transformation in *E. coli* (such as *rodZ*) and a few known competence gene homologs, such as *ybaV* and *yhiR* (Finkel and Kolter, 2001; Palchevskiy and Finkel, 2006), but do not include *rpoS* and other genes related to the RpoS-dependent mechanism (Zhang et al., 2012). Overall, these results point toward an unknown, complex mechanism of phage-induced, high-frequency HPTT that may partly share the pathway of natural transformation.

### HPTT Between Natural *E. coli* Strains

To further assess the generality and variety of HPTT in *E. coli* strains, natural strains (the aforementioned ECOR strains) were used in a study of HPTT. Several combinations of ECOR strains were co-cultured in liquid media, resulting in DNase-sensitive horizontal transfer of natural antibiotic resistance genes (Matsumoto et al., 2016a,b). Plasmid isolation from these new transformants demonstrated horizontal plasmid transfer between ECOR strains (Matsumoto et al., 2016a,b). Simple PT experiments using the same ECOR strains revealed that HPTT occurs much more frequently (10^−6^−10^−8^) than simple PT (below 10^−10^) under the same culture conditions, which suggested that HPTT is unique and effective. Moreover, we discovered that 6 of 12 combinations of the ECOR strains, some of which produce no plaque-forming phages (Shibata et al., 2014b), exhibited DNase-sensitive gene transfer, leading us to suspect that HPTT is rather common in natural *E. coli* strains. Overall, these data suggest that some phage- and conjugation-free transformation mechanism(s) also naturally exist in some *E. coli* strains and that HPTT of antibiotic-resistant natural plasmids (such as plasmids of the ECOR24 strain: Accession Nos. AB905284 and AB905285) can be a pathway for producing multidrug-resistant natural *E. coli* cells.

## Possible Mechanisms and Feasibility of PT and HPTT in *E. coli* in the Environment

Examples of PT and freeze–thaw-induced and low-frequency HPTT introduced in this mini-review are probably more related to the pore-forming mechanism than the competence gene-dependent mechanism because foods and natural waters often contain mM levels of Ca^2+^ and Mg^2+^ ions (Baur et al., 1996, Bauer et al., 1999; Maeda et al., 2003), and the biofilm environment supply living cells with the content of dead cells, including divalent metal ions and transformable plasmid DNA. As we described previously (Maeda et al., 2006), an SA biofilm (diameter, 10–12 mm; thickness, 0.5–0.8 mm) contains approximately 2–5 × 10^9^ cells. In addition, gut bacteria in mammals generally amount to approximately 10^11^ cells/g (Zoetendal et al., 2004; Sekirov et al., 2010). Considering the enormous scale of the environment, even transformation frequencies of 10^−9^−10^−10^ cannot be underestimated as they will have an impact on the bacteria populations.

High-frequency HPTT described in this article may involve not only the pore-forming mechanism but also a part of the competence gene functions and possibly another unknown mechanism, as mentioned above. Because bacteriophages are one of the most abundant organisms in the biosphere and ubiquitous in the environment (Clokie et al., 2011), phage-induced HPTT is also considered to be feasible in the environment, as well as ordinary transduction and other phage-derived ways of HGT, e.g., gene transfer agents (Lang et al., 2012).

## Conclusion and Perspective

Overall, our results and related previous data indicate that multiple mechanisms induce transformation-type HGT in *E. coli* based on various environmental and cellular circumstances such as the nature of the media (e.g., water and food), variable temperature from sub-zero to ∼40°C, high cell density in biofilms, and varying genetic backgrounds of the strains involved. The contribution of transformation-type HGT to genetic dynamics in the environment may be underestimated (Bushman, 2002; Thomas and Nielsen, 2005), and our studies indicate that HPTT in *E. coli* occurs at substantial transfer frequencies (10^−5^−10^−10^) under the conditions that can be feasibly encountered in the environment. Therefore, transformation-type HGT can contribute to the spread of antibiotic resistance genes and emergence of multidrug-resistant bacteria in the real environment outside laboratories. Further studies are required to understand the precise role and contribution of transformation-type HGT in spreading antibiotic resistance.

## Author Contributions

HH, ES, and SM wrote the paper.

## Conflict of Interest Statement

The authors declare that the research was conducted in the absence of any commercial or financial relationships that could be construed as a potential conflict of interest.
